# Defining the structural basis for human alloantibody binding to human leukocyte antigen allele HLA-A*11:01

**DOI:** 10.1038/s41467-019-08790-1

**Published:** 2019-02-21

**Authors:** Yue Gu, Yee Hwa Wong, Chong Wai Liew, Conrad E. Z. Chan, Tanusya M. Murali, Jiawei Yap, Chien Tei Too, Kiren Purushotorman, Maryam Hamidinia, Abbas El Sahili, Angeline T. H. Goh, Rachel Z. C. Teo, Kathryn J. Wood, Brendon J. Hanson, Nicholas R. J. Gascoigne, Julien Lescar, Anantharaman Vathsala, Paul A. MacAry

**Affiliations:** 1 Department of Microbiology and Immunology, Yong Loo Lin School of Medicine, National University of Singapore, Singapore, Singapore; 2 NUS Graduate School for Integrative Sciences and Engineering (NGS), National University of Singapore, Singapore, Singapore; 3 Immunology Programme, Yong Loo Lin School of Medicine, National University of Singapore, Singapore, Singapore; 4 School of Biological Sciences, Nanyang Technological University, Singapore, Singapore; 5 NTU Institute of Structural Biology, Nanyang Technological University, Singapore, Singapore; 6 DSO National Laboratories, Singapore, Singapore; 7 National University Centre for Organ Transplantation (NUCOT), National University Hospital, Singapore, Singapore; 8 Transplantation Research Immunology Group, Nuffield Department of Surgical Sciences, University of Oxford, Oxford, UK; 9 Division of Nephrology, Department of Medicine, Yong Loo Lin School of Medicine, National University of Singapore, Singapore, Singapore

## Abstract

Our understanding of the conformational and electrostatic determinants that underlie targeting of human leukocyte antigens (HLA) by anti-HLA alloantibodies is principally based upon in silico modelling. Here we provide a biochemical/biophysical and functional characterization of a human monoclonal alloantibody specific for a common HLA type, HLA-A*11:01. We present a 2.4 Å resolution map of the binding interface of this antibody on HLA-A*11:01 and compare the structural determinants with those utilized by T-cell receptor (TCR), killer-cell immunoglobulin-like receptor (KIR) and CD8 on the same molecule. These data provide a mechanistic insight into the paratope−epitope relationship between an alloantibody and its target HLA molecule in a biological context where other immune receptors are concomitantly engaged. This has important implications for our interpretation of serologic binding patterns of anti-HLA antibodies in sensitized individuals and thus, for the biology of human alloresponses.

## Abstract (web summary)

Anti-human leukocyte antigen (HLA) antibodies are important mediators of alloresponses, but structural insights on antibody:HLA interaction are still lacking. Here the authors provide a 2.4 Å structure of antibody:HLA complex, and also analyse HLA features important for other HLA-interacting molecules, to enhance our understanding of alloimmunity.

## Introduction

The development of alloantibody responses targeting human HLA molecules can be triggered by sensitization events that include blood transfusion, pregnancy or transplantation^1^. Anti-HLA alloantibody responses exhibit a broad range of overlapping reactivities based on the diversity of HLA alleles found in the human genome combined with the high degree of sequence homology between alleles^2^. The principal methodology employed to assess allosera allele reactivity is solid-phase multiplex binding assay^3^. Our current understanding of the structural determinants of an alloantibody response rely upon analyzing antibody binding patterns for HLA alleles in human serum combined with binding-site prediction algorithms that utilize HLA amino acid sequence alignment and/or stereochemical modeling^4,5^. The predicted binding motifs for alloantibodies determined using these methods are termed eplets. Eplets are defined as small patches of one or more polymorphic residue(s) within a radius of 3–3.5 Å, that differentiate the allele specificity of alloantibody responses^6^. An extensive eplet registry has been established using training data from allosera eluates preabsorbed on single HLA-expressing mammalian cell lines, rodent anti-HLA monoclonal antibodies and antibodies derived from Epstein−Barr virus transformed B-cell lines (EBV-BCLs)^4,7,8^. The principal weakness of this approach is that it does not define the true epitope of an alloantibody on HLA. A high-resolution structural footprint for a human anti-HLA alloantibody paratope−epitope interaction has not been previously reported.

The lack of structural data on the fine-specificity and related function(s) of human monoclonal anti-HLA alloantibodies potentially complicates the development of prognostic assays and associated clinical countermeasures in solid-organ transplantation^4,9,10^. For example, alloantibodies targeting donor-specific HLA are proposed to drive the inflammatory response that underlies long-term graft dysfunction and rejection^1,11,12^. An antibody binding to an HLA molecule on grafted tissues can result in the activation and deposition of complement components or direct immune effector cells expressing Fc-receptors to attack the graft. These activities can be influenced by the specificity, affinity, stereochemistry and subclass of the alloantibody^12–14^. Moreover, most chronic rejection responses occur within a time-frame where reconstitution of the transplant recipients’ immune cellular components has been initiated^15^. Under these circumstances, the stoichiometry of alloantibody binding to HLA may be complicated by cells binding to the same HLA molecules through other immune receptors such as TCR, KIR and CD8.

In this study, we report the development of an anti-HLA-A*11:01 human monoclonal alloantibody 2E3. We show that the pattern of allele specificity of 2E3 corresponds to that of naturally occurring polyclonal allosera described previously from other human donors^16,17^. We report the presence of antibodies derived from the same germline sequences as 2E3 in an HLA-sensitized individual. We present a 2.4 Å structure of 2E3 complexed with HLA-A*11:01 and compare this footprint with known binding sites for TCR, KIR and CD8 on the same molecule. We show that an eplet prediction algorithm accurately identifies a key residue (Asp90) that forms part of a larger epitope on the lateral surface of the HLA molecule and that this epitope does not occlude the binding sites for TCR, KIR or CD8. We present a biophysical analysis of 2E3 that details its binding affinity and on/off rates for HLA-A*11:01. Finally, we engineer recombinant human IgG1, IgG2, IgG3 and IgG4 subclass variants of 2E3 and compare their complement-dependent cytotoxicity (CDC) and antibody-dependent cell-mediated cytotoxicity (ADCC) activity on HLA-A*11:01 expressing target cell lines. We show that IgG1/3 induce significantly higher levels of CDC/ADCC and that IgG4 has low or negligible activity in both assays. These data represent a detailed analysis of the immunological, biochemical and biophysical properties of an HLA-specific, human monoclonal antibody and constitute the first step in addressing a significant gap that currently exists in our understanding of the fundamental biology of human alloantibodies.

## Results

### The allele reactivity of an anti-HLA-A*11:01 human antibody

We isolated a recombinant monoclonal antibody termed 2E3 from a human phage-Fab library by panning against recombinant HLA-A*11:01 (see Supplementary Fig. 1a). The antibody was expressed as a recombinant human IgG1 and an antigen-binding fragment (Fab), and the specificity for HLA-A*11:01 confirmed by immunoblot (see Supplementary Fig. 1b). A thorough biophysical analysis of the interaction between our human alloantibody and HLA-A*11:01 was conducted using quartz crystal microbalance (QCM) technology. The association and dissociation rate constants (*k*_a_ and *k*_d_) of the 2E3-IgG1-A*11:01 interaction were determined to be 1.04 × 10^5^ M^−1^ s^−1^ and 3.3 × 10^–4^ s^−1^ respectively by single cycle kinetics (SCK). The dissociation equilibrium constant (*K*_D_) was thus calculated to be 1.03 × 10^−8^ M (see Supplementary Fig. 1c). The *K*_D_ of 2E3-IgG1-A*11:01 was 0.16 × 10^–9^ M when estimated by enzyme-linked immunosorbent assay (ELISA; see Supplementary Fig. 1d). Both values may diverge from the actual, given the challenges associated with accurately measuring high affinity interactions. The true *K*_D_ is likely to be in the nM range taking both QCM and ELISA results into consideration.

**Fig. 1:**
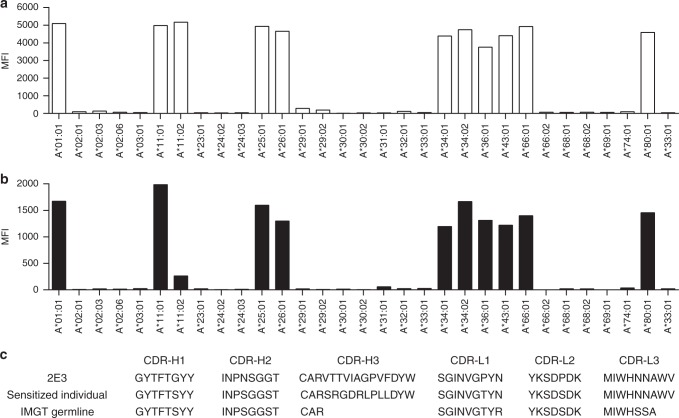
Allele reactivity and antibody CDR sequence of human monoclonal alloantibody 2E3. **a** The HLA-A allele reactivity of serum from a renal allotransplant recipient was assessed by OL-SAB. **b** The allele reactivity of 2E3-IgG1 was assessed at 1 μg mL^−1^ by OL-SAB and overlaps with that of the renal transplant recipient. **c** Heavy and light chain CDR amino acid sequences were compared between 2E3, a transplant recipient B-cell clone, and the corresponding germline sequences from IMGT®. The source data underlying Fig. 1a, b are provided as a Source Data file. CDR complementarity-determining region, OL-SAB One Lambda LABScreen Single Antigen beads, HLA human leukocyte antigens

We compared the allele reactivity of 2E3 with alloserum derived from a renal transplant recipient known to be making an alloantibody response against HLA-A*11:01. HLA allele reactivity was assessed using a clinical-grade alloantibody monitoring assay, namely the One Lambda LABScreen Single Antigen beads (OL-SAB) for HLA Class I. The observed allele reactivity pattern in the patient serum is shown (Fig. 1a). This pattern is comparable to that reported in other studies on HLA-A*11:01 sensitized individuals^16,17^. The isolated antibody termed 2E3 has an allele reactivity pattern that matches that observed in the sensitized individuals’ sera. Specifically, 2E3 was found to be reactive to A*01:01, A*11:01, A*25:01, A*26:01, A*34:01, A*34:02, A*36:01, A*43:01, A*66:01, A*80:01 and B*73:01 respectively. This pattern was identified using the OL-SAB, then confirmed by analyzing binding activity by flow cytometry on HLA-typed EBV-BCLs (Fig. 1b, Supplementary Fig. 2, and Supplementary Table 1).

Next-generation sequencing (NGS) of B-cell clones (see Supplementary Fig. 3) in the transplant recipient revealed the presence of both antibody heavy and light chains that are derived from the same recombination of germline sequences as that of 2E3. The complementarity-determining region (CDR) sequences of the transplant recipient antibody with the closest matches to 2E3 are listed in Fig. 1c. When compared to 2E3, a high degree of amino acid sequence similarity was observed at heavy chain CDR1 (87.5% identity), CDR2 (62.5% identity), and all light chain CDRs (92% identity). Given the high resemblance in both allele reactivity and CDR sequences, we can conclude that 2E3 is an antibody that can be developed during an alloresponse.

**Fig. 2:**
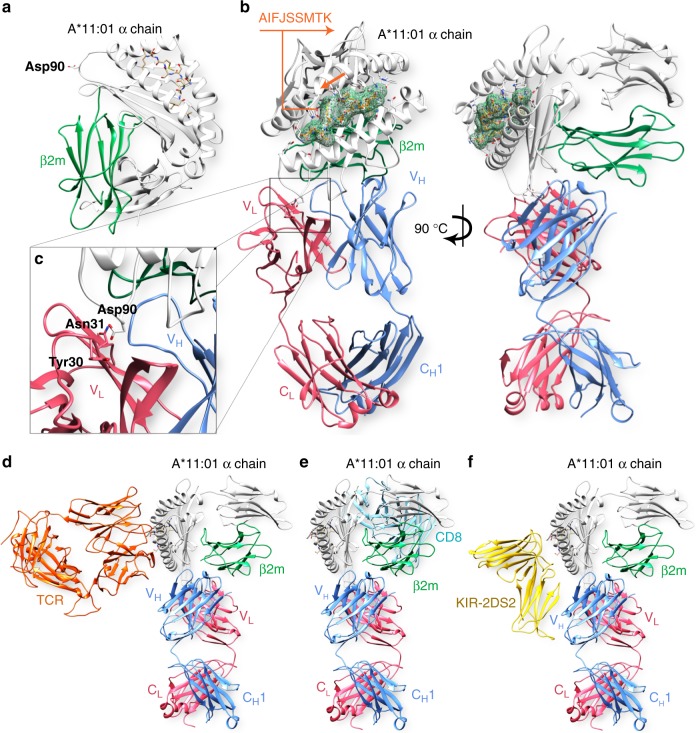
Key features of the binding interface between 2E3-Fab and HLA-A*11:01. **a** Aspartic acid at position 90 (Asp90) is the predicted eplet for 2E3 and is highlighted on the structure of A*11:01 (PDB 2HN7). **b** The structure of 2E3-Fab complexed with HLA-A*11:01 monomer was solved by X-ray crystallography at 2.4 Å. Fab fragment of 2E3 consists of heavy chain (blue ribbon) and light chain shown (pink ribbon). It interacts with refolded HLA-A*11:01 which comprises of α chain shown in white, β2m shown in green and a peptide shown as mesh. **c** Magnified view of the interaction between 2E3-Fab and HLA-A*11:01 near the predicted eplet (Asp90). The Asn31 of 2E3 light chain variable region interacts with Asp90 of HLA α chain. **d** View of interaction interface between 2E3-Fab and HLA-A*11:01, when KIR2DS2 (PDB 4N8V), **e** TCR (PDB 5WKH), and **f** CD8 (PDB 1AKJ) are bound to the same HLA molecule. HLA human leukocyte antigens

Analysis of 2E3 allele reactivity by eplet prediction from the HLA epitope registry identified the aspartic acid at position 90 (Asp90) of the HLA α chain as the putative eplet for this alloantibody (Fig. 2a). This also matches the eplet predicted for the patients sera and that identified in previous reports on HLA-A*11:01 sensitized individuals^16,17^. Taken together, these data indicate that 2E3 is a human alloantibody that targets a well-defined immune determinant on HLA-A*11:01.

### A high-resolution structural footprint of 2E3 on HLA-A*11:01

The crystal structure of 2E3-Fab interacting with refolded recombinant HLA-A*11:01 monomers was solved by X-ray crystallography to 2.4 Å (Fig. 2b, Table 1). As predicted, the resolved epitope includes the eplet defined as amino acid residue Asp90 of the HLA α chain (Fig. 2c). This forms a hydrogen bond with residue Asn31 and nonbonded contact with Tyr30 of 2E3’s light chain CDR1. 2E3 mainly binds to HLA α chain and partially interacts with beta-2-microglobulin (β2m; see Supplementary Table 2), forming a footprint of 1020.5 Å^2^ on the HLA surface. Binding of 2E3 induces a slight conformational change in HLA α chain residues 16–19 (see Supplementary Fig. 4).

**Table 1:** Crystallographic data and refinement statistics

PDB code	6ID4
Data collection	
Beamline	Proxima 2A, SOLEIL
Space group	C2
Cell parameters:	
*a*, *b*, *c* (Å)	124.56, 215.33, 80.98
*α*, *β*, *γ* (°)	90.00, 92.96, 90.00
Resolution (Å)	45.78–2.40 (2.46–2.40)
No. of measured reflections	233,036 (31,084)
No. of unique reflections	79,956 (12,545)
* R*_merge_	0.08 (0.51)
* CC*_1/2_	0.98 (0.56)
Average I/*σ* (I)	6.2 (1.36)
Completeness (%)	96.4 (93.6)
Multiplicity	2.9 (2.5)
Refinement	
Resolution (Å)	2.4
No. reflections	79,940
* R*_work_ / *R*_free_ (%)	19.7/24.6
No. atoms/*B*-factors	
Protein atoms	12,761/61.5
Water molecules	808/54.5
PEG	35/54.6
Glycerol	30/61.5
Correlation coefficient Fo/Fc	0.94
R.m.s. deviation	
Bond lengths (Å)	0.010
Bond angles (°)	1.23
Ramachandran plot (%)	
Favored	96.5
Allowed	3.5
Outlier	0

*PEG* polyethylene glycol

To simulate the interactions between a T cell, 2E3 and HLA, the structures of an A*11:01-recognizing TCR (PDB 5WKH) and CD8 (PDB 1AKJ) were superimposed onto the 2E3-A*11:01 complex (Fig. 2d, e, Supplementary Fig. 5a, b). The structure of KIR2DS2 (PDB 4N8V) was also superimposed onto our complex to mimic the interaction between natural killer cells (NK cells) and 2E3-bound HLA-A*11:01 molecules (Fig. 2f, Supplementary Fig. 5c).

### A structural perspective of 2E3 allele specificity

**Fig. 3:**
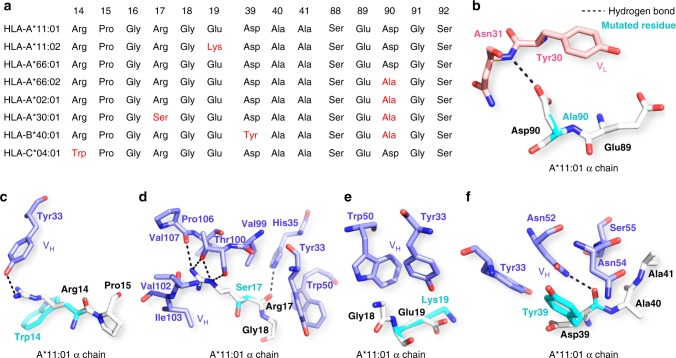
Structural analysis of 2E3 allele specificity. **a** Amino acid sequence alignment of HLA-A*11:01 with selected HLA alleles at the 2E3 epitope. **b** Magnified view of the interaction between HLA α chain Asp90 and 2E3 V_L_. Asp90 was duplicated and mutated to Ala90 (cyan). **c** Magnified view of the interaction between HLA α chain Arg14 and 2E3 V_H_. Arg14 was duplicated and mutated to Trp14 (cyan). **d** Magnified view of the interaction between HLA α chain Arg17 and 2E3 V_H_. Arg17 was duplicated and mutated to Ser17 (cyan). **e** Magnified view of the interaction between HLA α chain Glu19 and 2E3 V_H_. Glu19 was duplicated and mutated to Lys19 (cyan). **f** Magnified view of the interaction between HLA α chain Asp39 and 2E3 V_H_. Asp39 was duplicated and mutated to Tyr39 (cyan). HLA human leukocyte antigens

X-ray crystallography reveals that the epitope of 2E3 on A*11:01 α chain consists of three discontinuous segments. To investigate if this binding interface explains the allele reactivity of 2E3 as described in Fig. 1b, the protein sequences of all HLA Class I molecules included in the OL-SAB assay were aligned. Most of the alleles with mean fluorescence intensity (MFI) < 500 have Ala90 instead of Asp90. All residue differences in the 2E3 epitope are listed in Fig. 3a in the form of an amino acid sequence alignment of selected HLA Class I alleles. The structural effects of these residue changes were analyzed. The hydrogen bond between 2E3 light chain Asn31 and HLA α chain Asp90 was abrogated when Asp90 was replaced by Ala90 (Fig. 3b). The interaction between 2E3 heavy chain and HLA α chain was interrupted when positively charged Arg14 was replaced by hydrophobic Trp14 **(**Fig. 3c). Four hydrogen bonds between 2E3 heavy chain and HLA α chain were lost when Arg17 was changed to Ser17, which has a shorter side chain (Fig. 3d). Since no hydrogen bond was involved in the 2E3 heavy chain interaction with Glu19, 2E3 binding was not totally abrogated when negatively charged Glu19 was changed to positively charged Lys19 (Fig. 3e). The interaction between 2E3 heavy chain and HLA α chain was predicted to be affected when negatively charged Asp39 was replaced by hydrophobic Tyr39 (Fig. 3f).

### A functional comparison of IgG alloantibody subclasses

**Fig. 4:**
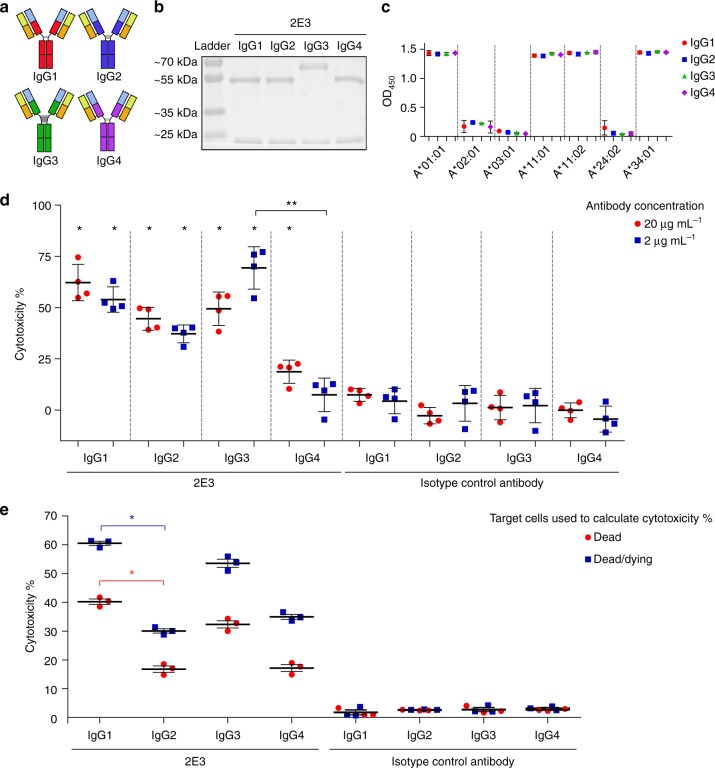
The influences of alloantibody IgG subclass on their effector function(s). **a** Schematic representation of the four human IgG subclasses. **b** Heavy and light chains of different subclasses of antibody 2E3 were resolved on polyacrylamide gel under reducing conditions. **c** Binding of four human IgG subclasses of alloantibody 2E3 at 1 μg mL^−1^ to refolded recombinant HLA monomers was tested by ELISA (mean ± s.d., *N* = 3 independent experiments). **d** CDC mediated by different IgG subclasses of 2E3 or respective isotype control antibodies (represented by target cell cytotoxicity) when incubated with complement (mean ± s.d., *N* = 4 independent experiments). *P = 0.0286, two-tailed Mann−Whitney test, compared with respective isotype control. **Gaussian approximated *P* = 0.0003 in Kruskal−Wallis test when compared all 2E3-treated groups, subsequently calculated to be statistically significant with *P* < 0.01 in Dunn’s multiple comparison test. **e** ADCC mediated by different IgG subclasses of 2E3 or respective isotype control antibodies (represented by target cell cytotoxicity) at a fixed monoclonal antibody concentration (25 μg mL^-1^) and effector:target ratio (4:1) when using NK cells as effector cells (mean ± s.e.m., *N* = 3 independent experiments). *Gaussian approximated *P* *=* 0.0156 when compared all 2E3-treated dead/dying cell populations, Gaussian approximated *P* = 0.0237 when compared all 2E3-treated dead cell population in Kruskal−Wallis test; subsequently calculated to be statistically significant with *P* < 0.05 in Dunn’s multiple comparison test. *P* = 0.1 (not significant), two-tailed Mann−Whitney test, when comparing each 2E3-treated group with the respective isotype control group. The source data underlying Fig. 4b–e are provided as a Source Data file. HLA human leukocyte antigens, ELISA enzyme-linked immunosorbent assay, CDC complement-dependent cytotoxicity, ADCC antibody-dependent cell-mediated cytotoxicity

Alloantibody 2E3 was engineered into the four principal human IgG subclasses while retaining the antibody variable region sequence plus the binding specificity for HLA-A*11:01 (Fig. 4a). IgG3 has a larger heavy chain of 60 kDa than the other three IgG subclasses due to its longer hinge region (Fig. 4b). The reactive alleles of IgG2, IgG3 and IgG4 subclasses of 2E3 were verified to be the same as the IgG1 counterpart by ELISA binding to refolded recombinant monomers (Fig. 4c). The CDC and ADCC of the four subclasses were compared using A*11:01 homozygous EBV-BCLs as targets. Both IgG1, IgG2 and IgG3 subclasses of 2E3 induced significant levels of CDC of target cells at a relatively low concentration of 2 μg mL^−1^. 2E3-IgG4 antibody only induced significant CDC at a high concentration of 20 μg mL^−1^ with less than 20% cell death (Fig. 4d). In summary, at low concentrations, the ability of 2E3-IgG1 or 2E3-IgG3 to induce cell death and injury was seven times higher than that of 2E3-IgG4 (Fig. 4d, Supplementary Fig. 6). Similarly, 2E3-IgG1 and 2E3-IgG3 were stronger than the other two IgG subclasses in mediating ADCC (Fig. 4e). Preincubation of 2E3-IgG1 or IgG3 at 25 μg mL^−1^ prior to the introduction of NK effector cells led to an approximately 50% increase in target cell death and injury compared to IgG2 and IgG4 (see Supplementary Fig. 7).

**Fig. 5:**
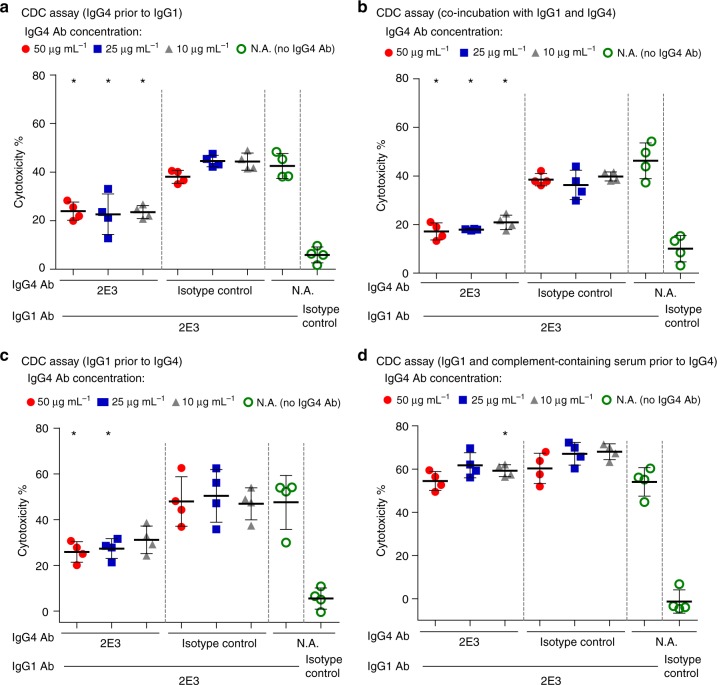
Inhibition of IgG1-induced CDC by IgG4. **a** CDC when target cells were sequentially incubated with IgG4 antibodies for 1 h, then IgG1 antibodies for 1 h, followed by complement serum for 3 h. **b** CDC when target cells were coincubated with IgG4 and IgG1 antibodies for 1 h, followed by complement serum for 3 h. **c** CDC when target cells were sequentially incubated with IgG1 antibodies for 1 h, then IgG4 antibodies for 1 h, followed by complement serum for 3 h. **d** CDC when target cells were coincubated with IgG1 antibodies and complement serum for 1 h, followed by addition of IgG4 antibodies and incubation for another 2 h. CDC result is represented by target cell cytotoxicity (mean ± s.d., *N* = 4 independent experiments). *P = 0.0286, two-tailed Mann−Whitney test, compared with respective isotype control. The source data underlying Fig. 5a–d are provided as a Source Data file. CDC complement-dependent cytotoxicity

Given the reduced complement-mediated killing by 2E3-IgG4, its ability to block CDC induced by 2E3-IgG1 was explored. We observed a significant reduction in the cytotoxicity in different conditions where 2E3-IgG4 was added prior to, at the same time, or after the IgG1 antibody (Fig. 5a–c). The statistical significance was lost when 2E3-IgG4 was added 1 h after coincubation with 2E3-IgG1 and complement-containing serum (Fig. 5d).

## Discussion

**Fig. 6:**
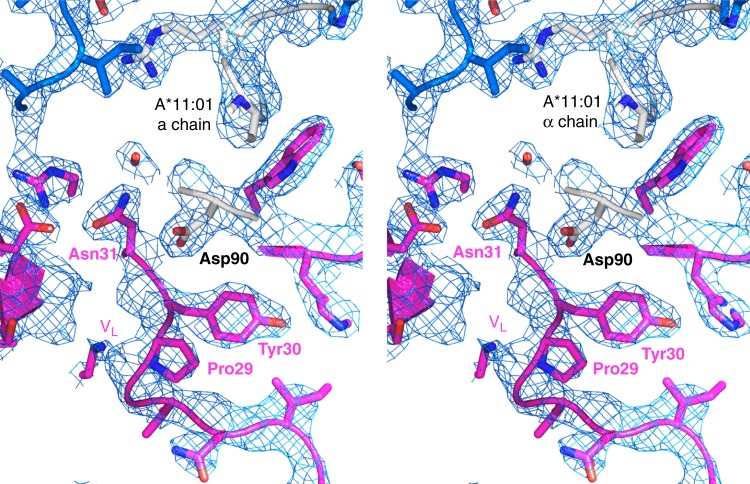
A 2Fo-Fc map showing the interaction between 2E3-Fab light chain and Asp90 of HLA-A*11:01. The map contour level is 1.0 sigma (0.1826 e/Å^3^)

In this study, we describe the generation and detailed structural characterization of a human monoclonal alloantibody binding to a lateral interface of a common Asian HLA allele, HLA-A*11:01. This monoclonal alloantibody is reactive to a pattern of HLA alleles that has been described for allosera from HLA-A*11:01 sensitized individuals. Previous studies have focused upon a determination of the pattern of allele specificity by multiplex solid-phase assay combined with in silico approaches to predict the likely eplets involved in antibody binding. In Fig. 2b, we present an alloantibody Fab-HLA complex crystal structure at a 2.4 Å resolution. This structural footprint reveals a lateral binding interface between 2E3-Fab and HLA-A*11:01. This binding site is distal to that employed by TCR on the same HLA molecule—it does not bind residues within or proximal to the peptide-binding groove^18^. We show that the CDR1 motif encoded on the light chain of 2E3 interacts closely with HLA α chain, including the algorithm-predicted eplet Asp90 (Fig. 6). The critical role of Asp90 was specifically delineated using sequence alignment of HLA-A*66:01 and HLA-A*66:02 (shown in Fig. 3a). The only amino acid differences between these two alleles are at position 90 and position 163, the latter being outside the defined epitope region for 2E3. The single residue change from Asp90 in A*66:01 to Ala90 in A*66:02 can completely abolish 2E3 binding to A*66:02.

We measured an interface area of 1020.5 Å^2^ between 2E3-Fab and HLA-A*11:01. All six CDRs of 2E3-Fab contributed to the hydrogen bonds, polar, or hydrophobic interactions with the target antigen. The HLA-A*11:01 α chain is highly involved (interface of 459.5 Å^2^ with Fab heavy chain and 258 Å^2^ with Fab light chain) compared to β2m (interface of 303 Å^2^ with Fab heavy chain). We observed that the 2E3-Fab heavy chain variable region binds partially to β2m of HLA, and that this may potentially confer cross-reactivity to most HLA class I alleles at high antibody concentrations. This lateral binding mode of 2E3 is not affected by the plasma membrane as this antibody can bind to the surfaces of HLA-A*11:01-positive EBV-BCLs (see Supplementary Fig. 2) and elicit downstream effector mechanisms (Fig. 4d, e) when HLA-A*11:01 is displayed in a physiological context.

We isolated antibody heavy and light chain sequences highly similar to that of 2E3 from an HLA-sensitized individual whose allosera binds to the HLA α chain Asp90. Most of the amino acid mismatches were found in the heavy chain CDR3 (CDR-H3; Fig. 1c). During VDJ recombination, a high degree of junctional diversity is introduced into CDR-H3 by the protein Artemis, terminal deoxynucleotidyl transferase (TdT), and exonucleases^19^. It is therefore expected that antibodies derived from the same germline will have CDR-H3 sequences that are unique to the individual.

It is clear from our structural analysis that the fine-specificity of 2E3 for HLA Class I alleles was defined by two residues, Asp90 and Arg14 on the HLA α chain (Fig. 3). We emphasize that the corresponding hydrogen-bonded residues, Asn31 (at CDR-L1) and Tyr33 (at CDR-H1) are both conserved in the transplant recipient (Fig. 1c). We therefore propose that 2E3-like naturally occurring alloantibodies should interact with HLA α chain in a similar way. The differences at CDR-H3 will have an impact on the interaction between patient allosera and HLA, especially the binding to β2m, but is unlikely to completely abrogate the binding or target a substantially different epitope.

We have modeled TCR and KIR2DS2 binding to A*11:01 onto our Fab-A*11:01 structure and predict little or no steric hindrance that would impact upon the simultaneous binding of both these receptors (see Supplementary Fig. 5a, c)^18,20^. We have also modeled the CD8αα homodimer bound to the Fab-A*11:01 structure and conclude that this should also be unimpeded (see Supplementary Fig. 5b)^21^. Although the binding sites for KIR, CD8 and TCR do not overlap with 2E3, it is worth considering that only the 50 kDa antibody Fab region was modeled on this structure. The potential steric effects on TCR, CD8 or KIR binding of a 150 kDa antibody molecule interacting with the same site cannot be predicted based on these analyses but should be significantly less than if 2E3 bound proximal to the peptide-binding groove.

It has been postulated that affinity may be a critical factor affecting alloantibody pathogenicity but detailed biophysical analyses of the binding of human alloantibodies to HLA are few in number^13^. In this study, we monitored the real-time dynamic interaction between 2E3-IgG1 and HLA-A*11:01 using third-generation biosensor QCM technology. Monoclonal alloantibody 2E3-IgG1 has a *K*_D_ in the 10^–8^ to 10^–9^ M range with a dissociation rate constant of 3.3×10^–4^ s^−1^. This *k*_d_ value is three times slower than that of any human EBV-BCL-derived monoclonal alloantibodies characterized in previous studies^13^. Previous studies have intimated an association between a slower *k*_d_ and stronger CDC activity, and by extension a potential link between alloantibody affinity, off rates and clinical outcomes^13^.

In transplant recipients, the activation of the classical complement pathway by polyclonal donor-specific alloantibodies (DSA) is proposed to be one of the major effector mechanisms that lead to graft injury and dysfunction and several studies have suggested that a DSA repertoire that is dominated by IgG3 is associated with significantly worse graft outcomes^14^. In addition, DSA responses can mediate rejection through other effector mechanisms that include the recruitment of immune effector cells, activation of endothelium and platelet aggregation^1,12^. The specific role of alloantibodies of a defined subclass is complicated by the presence of a heterogeneous mixture of specificities and isotypes in polyclonal DSA responses. We sought to provide a mechanistic insight into these associations by comparing the role of alloantibody IgG subclasses to recruit both complement and cellular effector mechanisms on HLA-A*11:01-expressing cells. We engineered alloantibody 2E3 into the four human IgG subclasses. Our functional characterization of these monoclonal antibodies revealed an overall trend that matched those observations on polyclonal allosera. Specifically, IgG3 and IgG1 were superior in mediating both ADCC and CDC. 2E3-IgG4 can inhibit the CDC induced by 2E3-IgG1 provided that complement-mediated killing has not been initiated. When the target cells are incubated with 2E3-IgG1 and complement serum for 1 h, IgG1-C1 complexes form on the cell surface. This would hinder the replacement of 2E3-IgG1 by subsequent additions of 2E3-IgG4, and thus the loss of the inhibitory effect of IgG4 (Fig. 5d). Our result suggests that there may be a way in which an IgG4 version of an alloantibody could be potentially developed to shield or occlude the epitope on HLA-A*11:01 by other antibodies.

These data represent the first step in deriving a detailed understanding of the biology of human anti-HLA alloantibodies. Based on these data, we propose that 2E3 represents an antibody that can be developed upon HLA sensitization, and provides insights on the antibody germline recombination that underlies a well-defined HLA allele specificity. This is important because the detection of polyclonal anti-HLA antibodies in allosera based on the current solid-phase assay is not enough to predict chronic rejection responses^22,23^. While many groups have described significantly worse graft outcomes in DSA-positive patients, others have shown that HLA-specific DSAs can be detected in long-term graft survivors with stable graft function^24^. This suggests that there is a degree of heterogeneity in the functionality and pathogenicity of an alloantibody response that cannot be assayed based on our current methods. Factors that include the specific epitope targeted, titer, affinity, and subclass have been proposed to influence the pathogenicity of an alloantibody response^12–14^. Thus, the repertoire of monoclonal human anti-HLA antibodies and structurally defined epitopes needs to expand to improve upon our predictive assays for triaging transplant recipients into those who should tolerate their graft in the long-term versus those who will require careful monitoring for the onset of chronic antibody-mediated rejection.

## Methods

### Study design

This study was designed to perform an in-depth structural, biochemical and biophysical characterization of a human monoclonal anti-HLA antibody. The pan-Class I HLA monoclonal antibody W6/32 (ATCC, cat. #HB-95) was included as a positive control for all binding assays to HLA antigens. In vitro cytotoxicity assays were conducted with the IgG subclass-engineered alloantibody and a human anti-dengue virus monoclonal antibody as the isotype control^25^.

### Ethics statement

Human peripheral blood was obtained upon informed consent from renal transplant patients (NHG DSRB Ref: 2015/00905) or healthy volunteers (NUS-IRB Reference Code: H-17-057). Study protocols are approved by National Healthcare Group (NHG) and National University of Singapore respectively. All procedures performed in studies involving human participants were in compliance with all relevant ethical regulations.

### HLA expression and purification

Briefly, plasmids encoding human β2m and the extracellular portion of α chain of defined HLA molecules were transformed into *Escherichia coli* BL21 for expression of the HLA subunits^26^. Protein inclusion bodies were extracted and refolding of peptide-HLA complexes performed in vitro by rapid dilution. Concentrated, dialyzed HLA proteins were biotinylated in vitro by recombinant biotin ligase (Avidity). Purification was performed by Fast Protein Liquid Chromatography followed by size exclusion chromatography (GE Healthcare Life Sciences).

### Phage-Fab display

A human Fab phage library (Humanyx Pte Ltd.) was used for panning against refolded recombinant HLA-A*11:01 based on a previously developed protocol^27^. Briefly, maxisorp immunotubes (Nunc) were coated with 20 μg mL^−1^ streptavidin at 4 °C overnight, blocked with 4% skim milk in Phosphate buffer saline (PBS), followed by an incubation with 20 μg mL^−1^ refolded recombinant HLA-A*11:01 at room temperature. The polyethylene glycol (PEG)-precipitated Humanyx Fab phage library was preabsorbed with streptavidin, blocked with 2% skim milk in PBS, and added to the antigen-coated immunotubes for binding over 1.5 h at room temperature. Immunotubes were then subjected to repeated washes with 4% skim milk in PBST (PBS with 0.05% Tween20), PBST, and PBS, and incubated with trypsin at 37 °C for 30 min. *E. coli* TG1 was incubated with eluted phages and cultured in 2xYT broth (BD Diagnostics) supplemented with 2% glucose till optical density at 600 nm (OD_600_) value reached 0.5. These *E. coli* TG1 was infected with M13KO7 helper phage by 30 min incubation at 37 °C, and then cultured overnight on 2xYT agar plates supplemented with 100 μg mL^−1^ carbenicillin and 25 μg mL^−1^ kanamycin at 30 °C. On the following day, phage-containing supernatants were harvested and Fab phages precipitated using PEG. An aliquot of the resulting PEG-precipitated Fab phages was used as the starting material for the following pan. Panning cycles were repeated four times to enrich for A*11:01-binding Fab phages.

### Polyclonal and monoclonal Fab phage ELISA

Single clones of Fab phages were selected based on ELISA screening against HLA-A*11:01^28^. Briefly, maxisorp plates were coated with HLA-A*11:01 at 4 μg mL^−1^ and blocked with 4% skim milk in PBST buffer. Streptavidin was coated as a negative control. These plates were then incubated with PEG-precipitated polyclonal Fab phages (diluted 10×) for 1 h. Subsequently, the plates were incubated with anti-M13-HRP secondary antibodies (GE Healthcare, cat. #27-9421-01, diluted 5000×) for 45 min followed by TMB substrate for color development. Plates were washed four times in PBST buffer between all incubation steps. H_2_SO_4_ was added to stop color development. The polyclonal Fab phages that gave the highest OD_450_ values for HLA-A*11:01 monomers but lowest OD_450_ values for streptavidin were used to infect *E. coli* TG1. The TG1 were cultured overnight on 2xYT agar plates supplemented with 100 μg mL^−1^ carbenicillin and 2% glucose. An estimate of 400 single colonies were individually cultured at 37 °C till OD_600_ value reached 0.5. These colonies were then infected with M13KO7 helper phage and cultured overnight in 2xYT broth supplemented with 100 μg mL^−1^ carbenicillin and 25 μg mL^−1^ kanamycin at 30 °C. Culture supernatants containing Fab phages were individually tested for binding to recombinant A*11:01 complexes by ELISA using the same protocol as polyclonal ELISA. Plasmids were extracted using QIAprep spin miniprep kit (QIAGEN) from colonies with high OD_450_.

### Whole antibody or antibody fragment expression

The variable region of the selected antibody heavy chain and full-length antibody light chain were cloned into their respective expression vectors. We utilized heavy chain vectors that contain the constant regions of human IgG1, IgG2, IgG3 or IgG4 for expression of full-length human IgG antibodies. Heavy chain vectors containing the constant region of mouse IgG1 was used for expression of mouse Fc chimeric antibodies. Heavy chain vectors containing human IgG1 C_H_1 region and a polyhistidine tag were used for expression of Fab fragments. Plasmids were transformed into *E. coli* TOP10 by heat shock and extracted using E.Z.N.A endo-free plasmid DNA mini kit (Omega Bio-Tek). Monoclonality was confirmed by sequencing (see Supplementary Table 3). Heavy and light chain plasmids were cotransfected into HEK293 cells using branched polyethylenimine (Sigma-Aldrich). Antibodies were purified from culture supernatants using protein G Sepharose 4 fast flow resin (GE Healthcare). Fab fragments were purified using cOmplete his-tag purification resin (Roche).

### Cell culture

EBV-BCLs are cultured with Roswell Park Memorial Institute (RPMI) media (GE Healthcare Life Sciences) + 10% fetal bovine serum (FBS; Gibco) + 1% penicillin/ streptomycin (Gibco) in T25 flasks and maintained at 37 °C with 5% CO_2_ in a cell incubator.

### Flow cytometry

0.5×10^6^ cells were incubated with 5 μg mL^−1^ of a mouse chimeric form of antibody 2E3 or control antibodies at 4 °C for 2 h in PBS supplemented with 3% FBS. After washing, cells were stained with 2 μg mL^−1^ AlexaFluor-488 conjugated goat-anti-mouse IgG antibodies (Life Technologies, cat. #A11029). All sample data were acquired using Attune NxT flow cytometer and data were analyzed using FlowJo VX software.

### Sandwich ELISA

Briefly, maxisorp plates were coated with streptavidin, blocked with 3% bovine serum albumin (BSA) in PBST buffer at room temperature and then incubated with biotinylated refolded recombinant peptide-HLA complexes at 4 °C overnight. Plates were then incubated with 1 μg mL^−1^ anti-HLA antibodies diluted in blocking buffer for 2 h. After that, each well was incubated with HRP-conjugated goat-anti-human IgG secondary antibody (Thermo Fisher Scientific, cat. #31413, diluted 10,000×) for 45 min, followed by incubation with TMB substrate for color development. Streptavidin coated wells with no HLA were included as negative controls. Plates were washed four times in PBST between all incubation steps. H_2_SO_4_ was added to stop color development. OD_450_ was recorded.

### Sodium dodecyl sulfate-polyacrylamide gel electrophoresis (SDS-PAGE)

To visualize protein sizes under reducing or nonreducing conditions, 2 μg protein was mixed with reducing or nonreducing dye, incubated at 95 °C for 10 min and loaded into each lane. Protein ladder (Thermo Fisher Scientific, cat. #26619) was loaded as a reference. Gel electrophoresis was carried out with a 12% polyacrylamide resolving gel. Gel was stained with Coomassie Brilliant Blue and background was de-stained by soaking in water.

### Western blot

Briefly, protein bands separated on SDS-PAGE were transferred to a methanol-activated polyvinylidene fluoride (PVDF) membrane. The membrane was blocked in 5% skim milk in PBST for 1 h. Subsequently, the membrane was incubated with 10 μg mL^−1^ primary antibody or Fab followed by incubation with goat-anti-human IgG-HRP (Thermo Fisher Scientific, cat. #31413, diluted 10,000×) or anti-his-HRP (Abcam, cat. #ab18184, diluted 1000×). Three washes for 5 min in PBST were conducted after each incubation step. WesternBright ECL (Advansta) was added for protein visualization.

### Single cycle kinetics

Antibody kinetics and affinity was measured using an Attana Cell A200 (Attana AB) at 25 °C. Attana LNB-carboxyl sensor chips were activated with sulfo-NHS/EDC and saturated with streptavidin. Ethanolamine was added to deactivate the chip surface. PBS was used as running buffer for subsequent injections. Biotinylated refolded recombinant HLA-A*11:01 was added to the experiment chip but not the reference chip by one injection of 4 μg mL^−1^ at 10 μL min^−1^ for 300 s. 2E3-IgG1 was injected at increasing concentrations ranging from 2.5 to 20 nM. Each injection lasted for 105 s at a flow rate of 20 μL min^−1^. Dissociation was monitored for 10 min after the last injection. Curve fitting and subsequent data analysis was performed by specialists from Attana AB.

### Affinity determination by ELISA

Briefly, a maxisorp plate was coated with 100 μL of streptavidin at a concentration of 4 μg mL^−1^ for 4 h at room temperature. Plate was washed four times with PBST and then blocked with 380 μL of 3% BSA in PBST for 2 h at room temperature. After blocking, plate was washed four times with PBST again and incubated with 150 μL of increasing concentrations of biotinylated antigen for 16 h at room temperature on shaker. Plate was then washed five times with PBST and incubated with 150 μL of increasing concentrations of primary antibody for 8 h at room temperature on shaker. After this the plate was washed five times with PBST and incubated with 85 μL of goat-anti-human IgG-HRP (Thermo Scientific, cat. #31419, diluted 10,000×) for 45 min at room temperature, protected from light. After five washes with PBST, 80 μL of TMB substrate was added to each well and color developed for 10 min, protected from light. Reaction was stopped with 80 μL of 1 M H_2_SO_4_ and OD_450_ was recorded.

### OL-SAB assay

Antibody 2E3-IgG1 (final concentration 1 μg mL^−1^) was prepared by 20× dilution in negative control serum (One Lambda Inc.). Diluted 2E3-IgG1 was tested using LABScreen® Single Antigen HLA Class I (One Lambda Inc.) by the Singapore Health Sciences Authority (HSA) laboratory that is internationally accredited for conducting solid-phase anti-HLA antibody detection assays. Transplant recipient serum was tested using the same platform.

### B-cell sequencing

Peripheral blood mononuclear cells from a kidney transplant recipient were resuspended at a concentration of 4×10^7^ cells per mL in staining buffer (PBS supplemented with 1% fetal bovine serum) and stained with mouse-anti-human CD27-AF647 (AbD Serotec, cat. #MCA775A647, 50× dilution), mouse-anti-human CD19-PB (AbD Serotec, cat. #MCA1904PB, 25× dilution) and goat-anti-human IgM-AF488 (Life Technologies, cat. #A11029, 25× dilution) for 1 h at 4 °C, protected from light. Cells were washed, resuspended, and stained with propidium iodide (Sigma-Aldrich, cat. #P4864-10ML) at a final concentration of 10 μg mL^−1^. Cells gated CD19^+^ were sorted on BD FACSAria Fusion (BD Biosciences). Gating strategy is provided in Supplementary Fig. 3.

Sorted B cells were resuspended in RNA extraction buffer (QuickExtract RNA Extraction Kit, Lucigen) at a ratio of 20,000 cells per 10 μL. cDNA was generated using the Maxima H Minus cDNA Synthesis Master Mix (Thermo Fisher Scientific) using 5 μL of extraction buffer per 10 μL reaction as per the manufacturer’s protocol. Two microliters of cDNA product was directly used in 20 μL reactions with Platinum Taq Master Mix as per the manufacturer’s protocol for separate amplification of the heavy and light chain variable regions. Illumina adaptor and barcode sequences added by further PCR with Q5 Hot Start Hi-Fidelity Master Mix and the PCR products purified with AMPure XP magnetic beads (see Supplementary Table 3). Purified PCR products were pooled in the same molar ratio as the number of B cells from each of the four samples, diluted to 8 pM and sequenced on the Illumina MiSeq with 2 × 300 bp kit with 25% PhiX spike in. Individual reads were separated by sample and analyzed with MIXCR^29^ to derive unique clonotypes based on CDR1 and 3 homology. Reads that comprise the clonotypes of interest (with matching V(D)J genes to Humanyx library antibody) were extracted and then used to generate consensus sequences of the entire variable heavy and light chains (SeqMan, DNAstar) for further analysis. The corresponding germline sequences were retrieved from IMGT, the international ImMunoGeneTics database®^30^.

### Crystallography, data collection and structure solution

Purified HLA-A*11:01 and 2E3-Fab were mixed to give a 1:1 stoichiometry and incubated at 4 °C for 2 h. The complex and unbound proteins were separated by size exclusion chromatography using a Superdex 200 16/600 (GE Healthcare). Complex was concentrated using an MWCO 50 kDa (Vivaspin) to a final concentration of 15 mg mL^−1^. Initial crystallization conditions were identified using a Mosquito (Art Robbins) crystallization robot at 20 °C using 96-well sitting drop format (a volume of 0.13 μL of protein was mixed with an equal volume of precipitant per drop). A hit was found using the PEGRx (Hampton Research) condition number 75 (2 % v/v 1,4-Dioxane, 0.1 M Tris pH 8.2, 20% w/v Polyethylene glycol 3,350). Plate shape crystals were reproduced using same crystallization buffer condition by mixing 1 μL of protein with 1 μL of buffer. Prior to data collection, crystals were briefly soaked in their respective precipitating solution supplemented with 25% (v/v) glycerol and rapidly frozen in liquid nitrogen.

X-ray diffraction data were collected at the beamline PROXIMA 2A at SOLEIL Synchrotron (France). This crystal diffracted to 2.4 Å. The data set was processed with XDS and XscALE^31,32^. The structure of the complex protein was determined by molecular replacement using PHASER-MR^33^ within the PHENIX software package^34^, and using the PDB code: 1W72 as search probe. A model for the 3D structure of the complex HLA-A*11:01 with 2E3-Fab was built iteratively at the computer graphics using COOT^35^, and refined using BUSTER^36^.

### CDC assay

Briefly, 0.05×10^6^ EBV-BCLs were incubated with 2 μg mL^−1^ or 20 μg mL^−1^ antibodies of interest at 37 °C for 1 h. In inhibition CDC assays, EBV-BCLs were incubated with the two types of antibodies sequentially for 1 h each at 37 °C. The IgG4 antibodies were titrated to a final concentration of 10 μg mL^−1^, 25 μg mL^−1^ or 50 μg mL^−1^. The IgG1 antibodies were added at a final concentration of 5 μg mL^−1^. This was followed by 3 h incubation with 10% baby rabbit complement serum (Cedarlane, cat. #CL3441) at 37 °C. Cells were then pelleted, washed in PBS, and stained with SytoxGreen and C12-Resazurin (Thermo Fisher Scientific, cat. #L34951) as per the manufacturer’s protocol. Cells were acquired with Attune NxT flow cytometer and data analyzed using FlowJo VX. SytoxGreen^high^ and Resorufin^low^ population was defined as dead cells. Gating strategy is provided in Supplementary Fig. 6a. Cytotoxicity% was determined as (experimental cell death – baseline cell death) / (maximum cell death – baseline cell death) × 100%.

### ADCC assay

Effector NK cells were cultured at 37 °C overnight in RPMI media (GE Healthcare Life Sciences) supplemented with 2000 units mL^−1^ IFN-α (Thermo Fisher Scientific, cat. #PHC4814). Target EBV-BCLs were stained with CFSE (Thermo Fisher Scientific, cat. #C34564) at 1000× dilution and cultured in RPMI media in 96-well V-bottom plates at 0.02×10^6^ cells per well and incubated with 25 μg mL^−1^ antibodies of interest at 37 °C for 1 h. NK cells were then added to EBV-BCLs at an effector:target ratio of 4:1 and incubated at 37 °C for 4 h. All cells were pelleted and stained with 20 μg mL^−1^ 7-AAD (Thermo Fisher Scientific, cat. #A1310). The CFSE^+^ 7-AAD^high^ population were defined as dead target cells while CFSE^+^ 7-AAD^mid^ population were defined as dying target cells. Gating strategy is provided in Supplementary Fig. 7a. Cytotoxicity% was determined as (experimental cell death – baseline cell death)/ (maximum cell death – baseline cell death) × 100%.

### Statistical analysis

Statistical analysis was performed using Graphpad Prism 5. Graphs with error bars were represented as mean ± s.d. or s.e.m. (*N* = 3 or 4). Two-tailed Mann−Whitney test was used to compare experimental (2E3-treated) group with the corresponding isotype control group. A Kruskal−Wallis test followed by Dunn’s multiple comparison test was used for comparison between different concentrations and/or subclasses. Statistical significance with a *P* value of <0.05 was labeled as * and *P* < 0.01 labeled as **.

### Reporting summary

Further information on experimental design is available in the Nature Research Reporting Summary linked to this article.

## Supplementary information

Supplementary Information

Reporting Summary

Source Data

## Data Availability

The atomic coordinates and structure factors of 2E3-HLA-A*11:01 have been deposited with the worldwide Protein Data Bank (wwPDB) under accession code 6ID4. The NGS data analyzed by MIXCR have been deposited with figshare under 10.6084/m9.figshare.7627484. A reporting summary for this article is available as a Supplementary Information file. The source data underlying Figs. 1a, b, 4b−e, 5a−d, and Supplementary Figs. 1b−d are provided as a Source Data file.
