# Supplementary material for: Defining the structural basis for human alloantibody binding to human leukocyte antigen allele HLA-A*11:01
Source: Nat Commun. 2019 Feb 21;10:893. doi: 10.1038/s41467-019-08790-1 (PMC6385295; doi:10.1038/s41467-019-08790-1)
Supplement: Supplementary file 1 — Supplementary Information [file 41467_2019_8790_MOESM1_ESM.pdf]

Supplementary Information for

Defining the structural basis for human alloantibody binding to human leukocyte antigen allele HLA-A*11:01

Gu *et al.*

Supplementary Figures

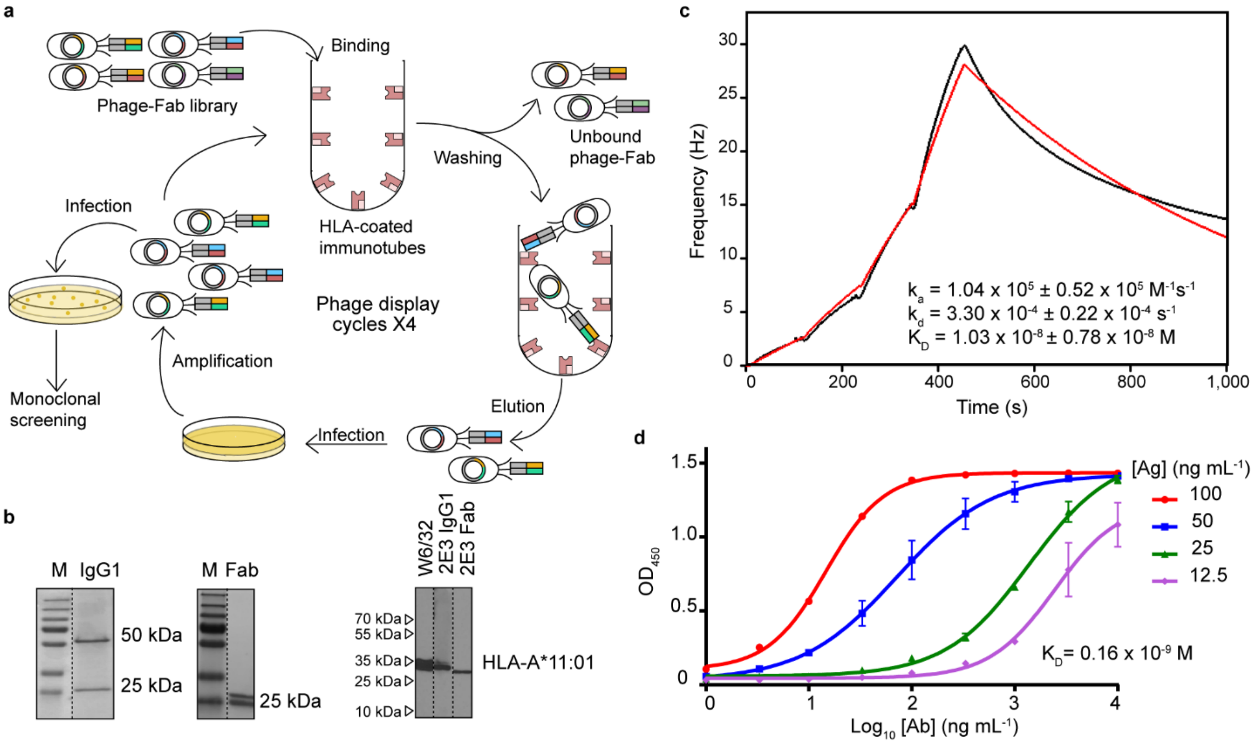

Supplementary Figure 1. Development and biophysical characterization of a human monoclonal alloantibody 2E3. **(a)** Schematic of the phage-Fab library panning process against refolded recombinant HLA-A*11:01. **(b)** Alloantibody 2E3 was expressed as a full-length IgG1, which consists of a 50 kDa antibody heavy chain and a 25 kDa antibody light chain when resolved on a reducing SDS-PAGE (left panel). A Fab fragment of 2E3 was expressed with the correct conformation consisting of one heavy chain and one light chain, both of approximately 25 kDa (middle panel). Immunoblot detection of both forms of 2E3 binding to HLA-A*11:01 α chain. Pan-HLA Class I-reactive monoclonal antibody W6/32 was used as a positive control (right panel). Protein ladders are labelled as M. **(c)** Single cycle kinetic study of 2E3-IgG1 was studied by QCM (mean ± s.e.m., N = 3, representative plot from one experiment is shown). Dissociation equilibrium constant of 2E3-IgG1 binding to HLA-A*11:01 was calculated to be 1.03 x 10^{-8} M. **(d)** Dissociation equilibrium constant of 2E3-IgG1 antibody binding to HLA-A*11:01 monomers was in the 10^{-9} M range when measured by ELISA (mean ± s.d., N = 3 independent experiments). The source data underlying **Supplementary Fig. 1b-d** are provided as a Source Data file.

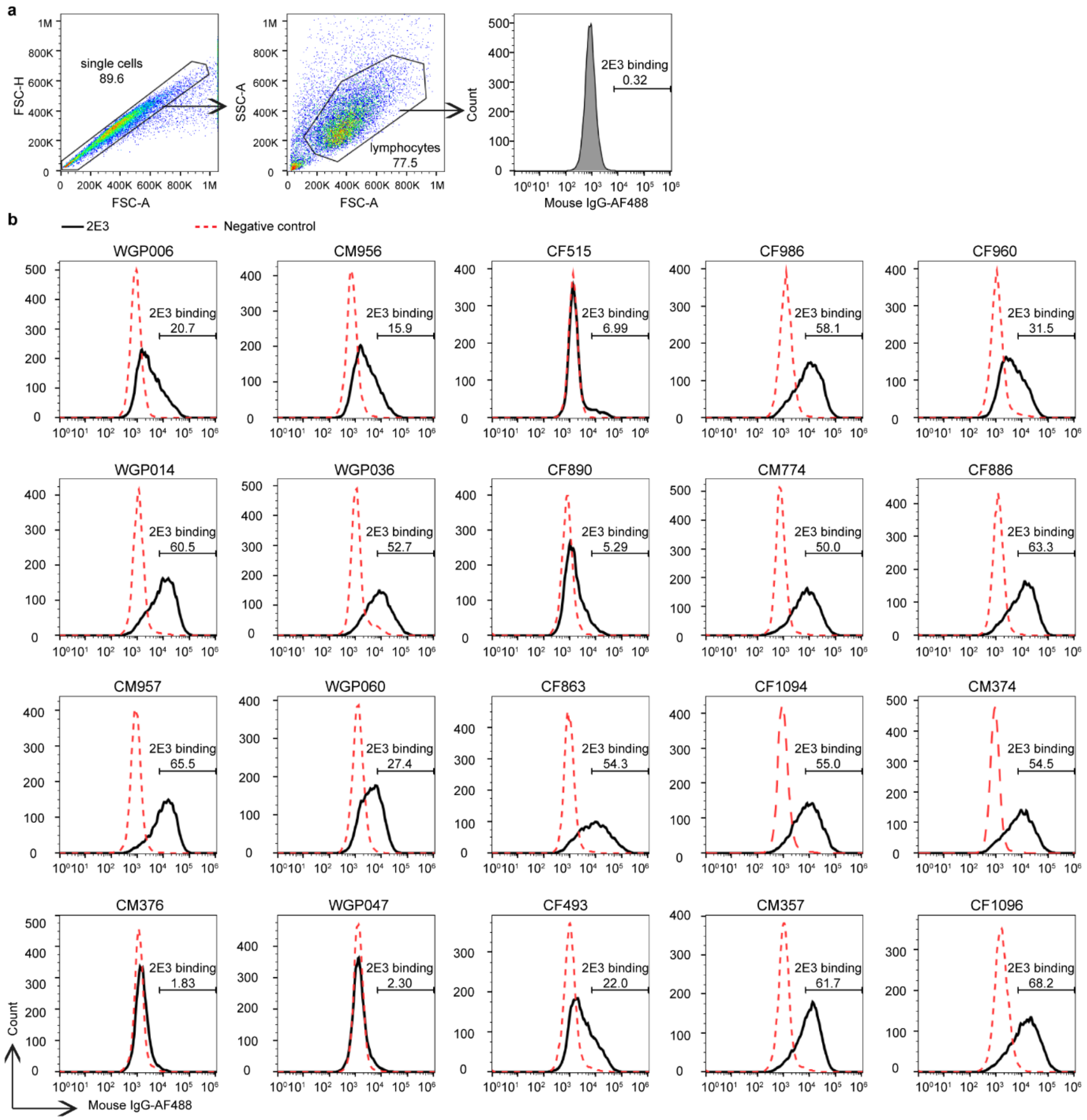

Supplementary Figure 2. Alloantibody 2E3 reactivity to EBV-BCLs measured by flow cytometry. **(a)** Gating strategy for one representative sample is shown. **(b)** Superimposed histogram of 2E3 binding to 20 different tissue-typed EBV-BCLs compared to negative control.

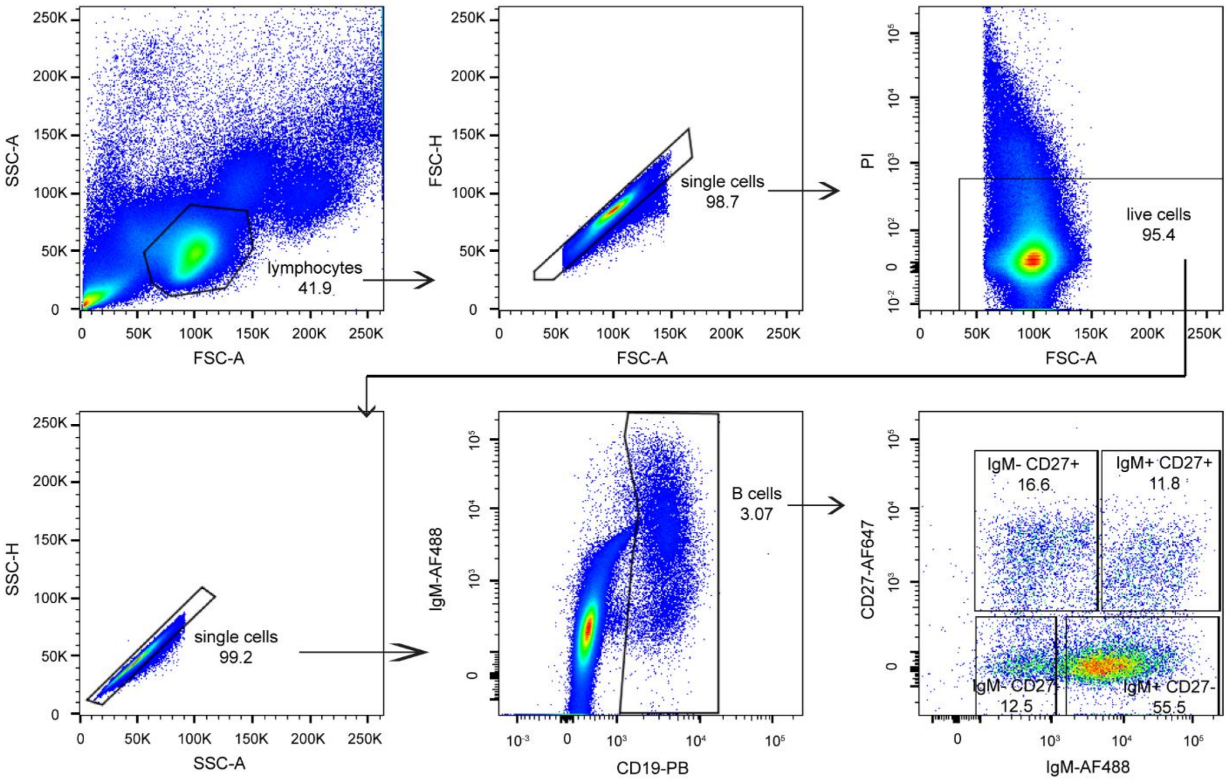

Supplementary Figure 3. Gating strategy used for cell sorting. CD19^{+} B cells of a transplant recipient were sorted from PBMCs into four different populations (IgM^{-} CD27^{+}, IgM^{+} CD27^{+}, IgM^{-} CD27^{-}, IgM^{+} CD27^{-}) using this gating strategy.

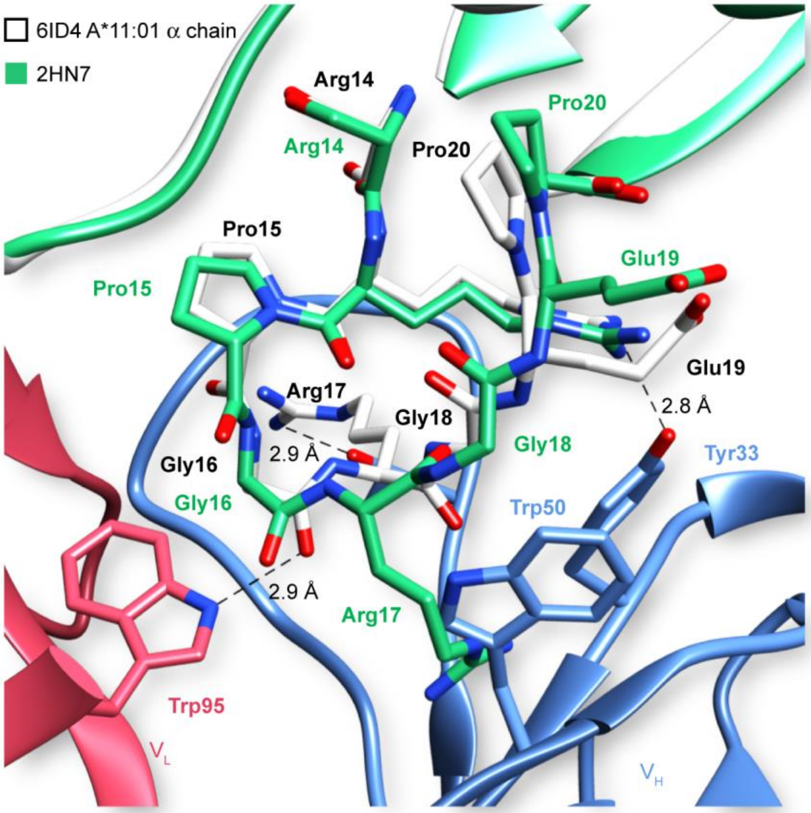

Supplementary Figure 4. Magnified view of amino acid residues 16-19 on HLA-A*11:01. A conformational change at residues 16-19 of the HLA α chain was observed when bound to 2E3 compared to the unbound HLA-A*11:01 monomer (PDB 2HN7).

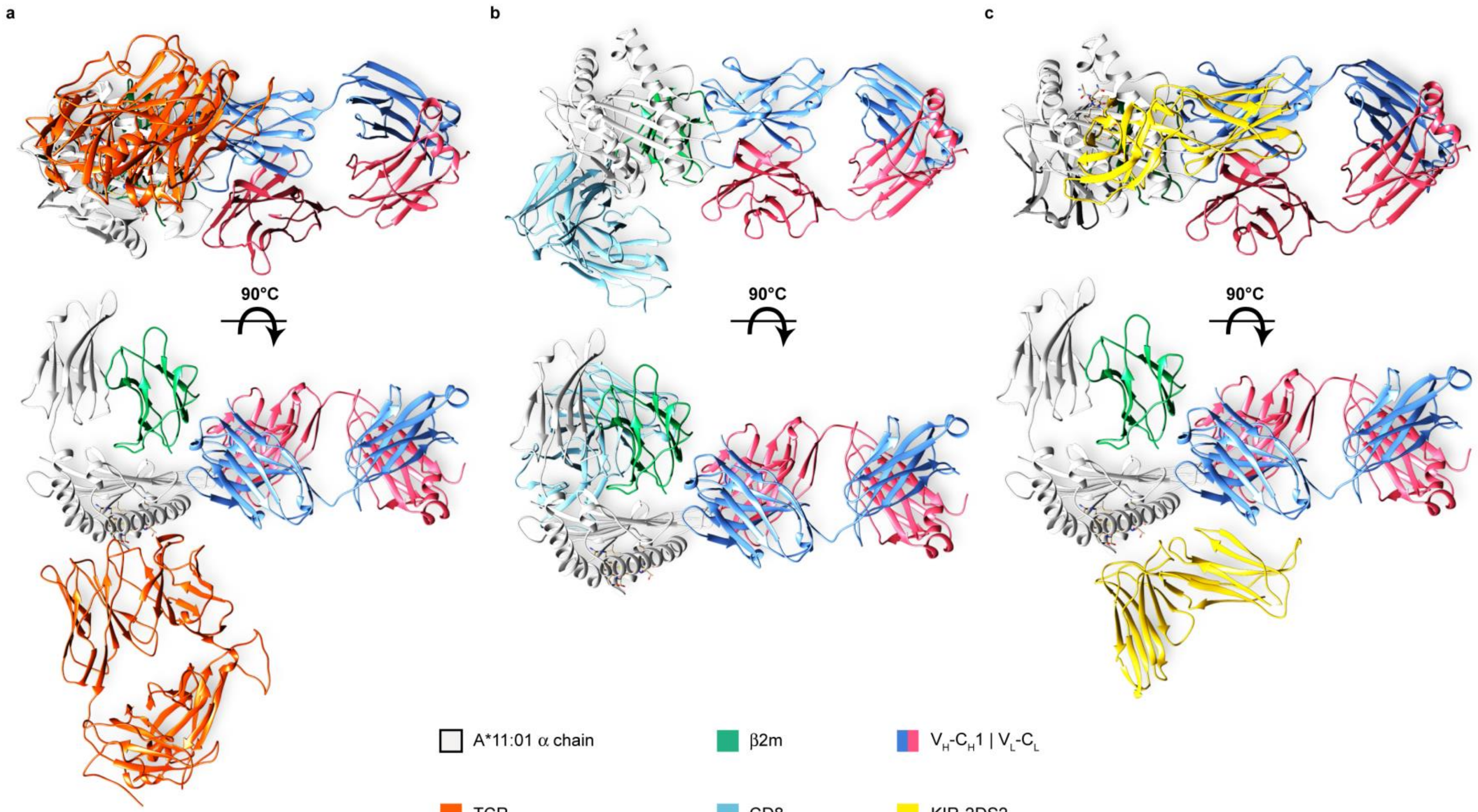

Supplementary Figure 5. Modelling of the binding interface between 2E3-Fab, HLA-A*11:01 and other cell surface receptors. The binding sites of the receptors **(a)** TCR, **(b)** CD8 and **(c)** KIR2DS2 on HLA-A has been previously resolved. These structures were superimposed to our 2E3-A*11:01 to compare the binding sites utilized by these receptors with our alloantibody 2E3. We can predict that there should be no occlusion of TCR, CD8 or KIR2DS2 binding sites by 2E3-Fab.

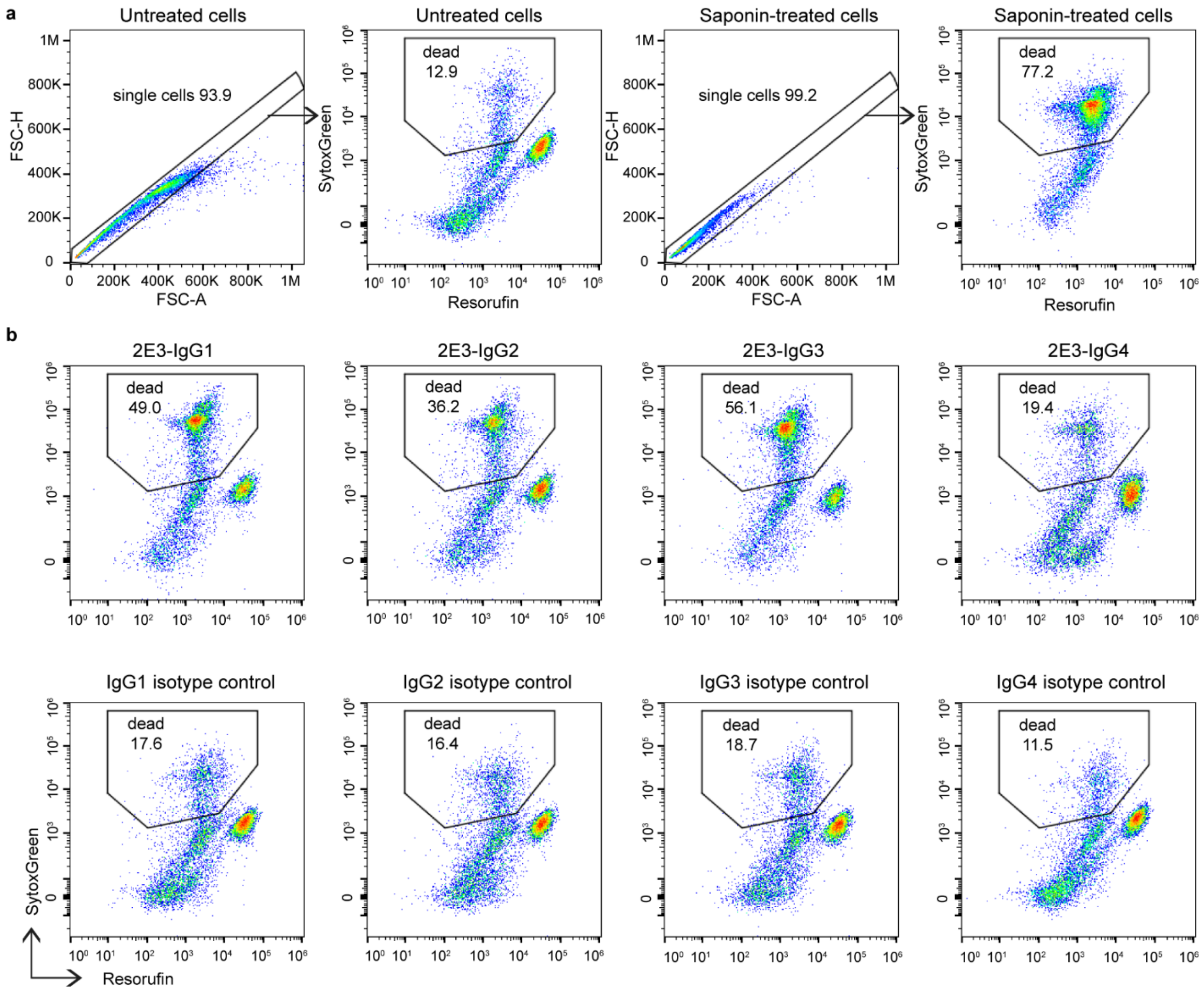

Supplementary Figure 6. CDC assay. **(a)** Gating strategies are shown for untreated cells and saponin-treated cells. The SytoxGreen^{high} Resorufin^{low} cell population was defined as the dead population. **(b)** Representative flow cytometry plots are shown for target cells treated with various antibody subclasses at 2 μg mL^{-1} in the CDC assay.

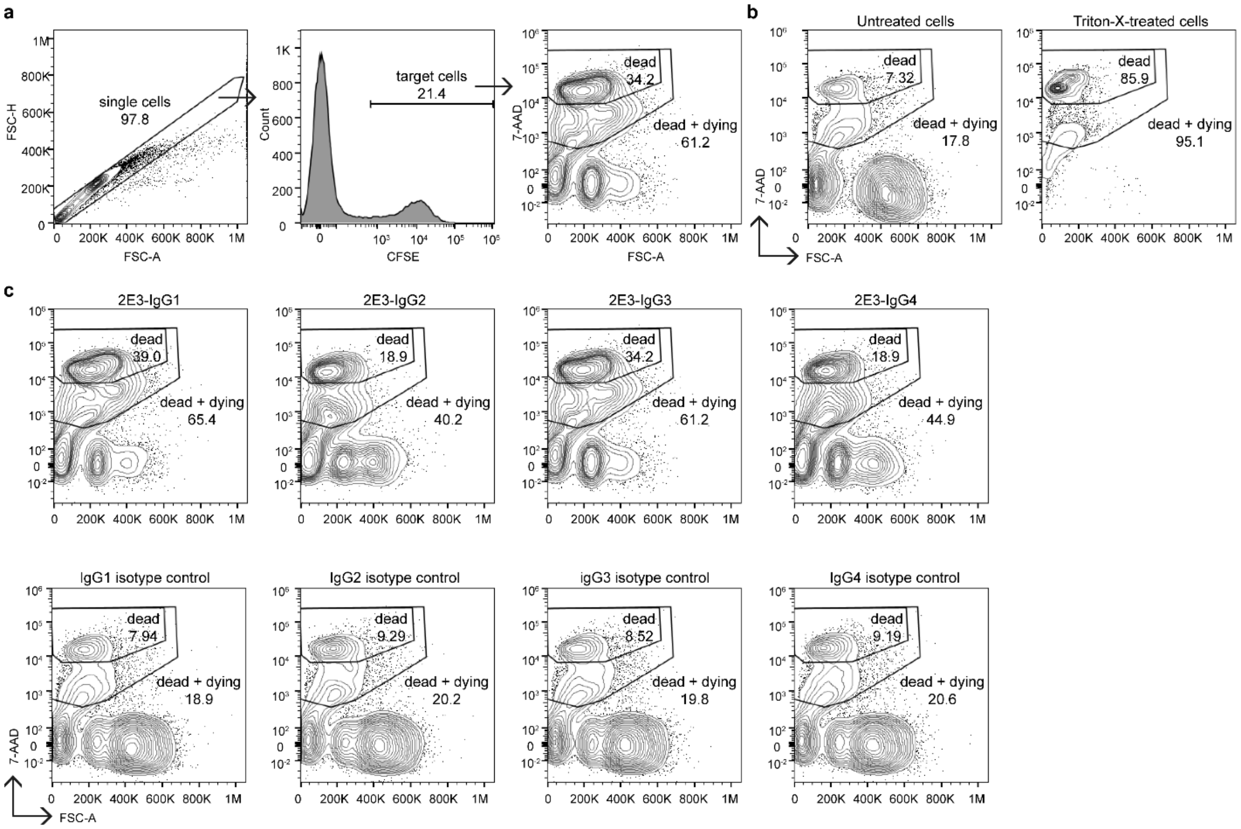

Supplementary Figure 7. ADCC assay. **(a)** Gating strategy of one representative sample is shown. Target cells were labelled with CFSE. Dead cells were gated as the FSC^{low} 7-AAD^{high} population. Dying cells were defined as the FSC^{low} 7-AAD^{mid} population. **(b)** Representative flow cytometry plots for untreated cells and Triton-X-treated cells. **(c)** Representative plots for target cells treated with different subclasses of antibodies in the ADCC assay.

Supplementary Tables

Supplementary Table 1. Class I HLA typing results of EBV-BCLs used for flow cytometry

EBV-BCL	HLA-A	HLA-A	HLA-B	HLA-B	HLA-C	HLA-C
WGP060	A*30:01	A*30:01	B*13:02	B*13:02	C*06:02	C*06:02
CM376	A*30:01	A*29:01	B*07:05	B*35:01	C*08:01	C*15:05
CF890	A*30:01	A*24:01	B*13:02	B*55:02	C*03:03	C*06:02
CF1096	A*11:01	A*26:01	B*46:01	B*40:01	C*07:02	C*01:02
CM357	A*02:01	A*26:01	B*40:01	B*39:01	C*07:02	C*15:02
CF986	A*02:01	A*02:01	B*40:01	B*51:01	C*07:02	C*15:02
CF960	A*02:03	A*02:03	B*38:02	B*38:02	C*07:02	C*07:02
CF863	A*11:01	A*11:01	B*51:01	B*55:02	C*14:02	C*01:02
CM374	A*33:03	A*11:01	B*15:02	B*39:01	C*08:02	C*07:02
CM774	A*11:01	A*03:01	B*13:01	B*35:03	C*04:01	C*03:04
CM956	A*02:03	A*02:07	B*39:01	B*51:02	C*07:02	C*15:02
CM957	A*11:01	A*11:02	B*13:01	B*27:04	C*03:04	C*12:02
CF493	A*31:01	A*24:02	B*40:01	B*35:01	C*07:02	C*03:03
CF515	A*26:01	A*24:02	B*40:06	B*55:02	C*12:03	C*08:01
CF886	A*11:01	A*11:02	B*18:01	B*38:02	C*07:02	C*15:02
CF1094	A*11:01	A*03:01	B*08:01	B*55:02	C*07:02	C*01:02
WGP006	A*02:01	A*02:07	B*48:01	B*46:01	C*01:02	C*08:01
WGP014	A*02:03	A*02:07	B*52:01	B*46:01	C*01:02	C*12:02
WGP036	A*02:03	A*26:01	B*40:01	B*40:06	C*03:04	C*07:02
WGP047	A*24:02	A*24:02	B*40:01	B*40:02	C*03:04	C*07:02

Supplementary Table 2. Key residues that form hydrogen bonds between 2E3-Fab and HLA-A*11:01

	Chain	Res No.	Res Name	Atom name		Chain	Res no.	Res name	Atom name	Distance
**1**	A	14	Arg	NH2	←→	H	33	Tyr	OH	2.83
**2**	A	17	Arg	O	←→	H	35	His	NE2	3.12
**3**	A	17	Arg	NE	←→	H	100	Thr	O	2.97
**4**	A	17	Arg	NE	←→	H	100	Thr	OG1	3.25
**5**	A	17	Arg	NH2	←→	H	100	Thr	OG1	2.88
**6**	A	17	Arg	NH2	←→	H	106	Pro	O	2.97
**7**	A	39	Asp	O	←→	H	52	Asn	ND2	2.80
**8**	A	16	Gly	O	←→	L	95	Trp	NE1	2.88
**9**	A	90	Asp	OD2	←→	L	31	Asn	N	3.10
**10**	B	36	Glu	OE2	←→	H	101	Thr	OG1	3.20

Supplementary Table 3. Primers used in the study

Name	Sequence	Usage
HX01F	AGCGGATAACAATTTCACACA	Sequence Fab light chain in pCES vector
pCES-LR	ACAATCCAGCGGCTGCCGTA	
pCES-HF	GGCGCGCCAATTCTATTTCAAG	Sequence Fab heavy chain in pCES vector
HX01R	TTTGTCGTCTTTCCAGACGTTAGT	
Seq01B	CGGATCTCTAGCGAATTCC	Sequence Ab heavy/ light chain in pFabTT5 vector
Seq03	CCTTTATTAGCCAGAGGT	
CKrevUMI	GTCAGATGTGTATAAGAGACAGNNNNNNGACAGATGGTGCAGCCACAGTTCG	cDNA generation
CLrevUMI	GTCAGATGTGTATAAGAGACAGNNNNNNGAGGAGGGYGGGAACAGAGTGAC	
IgGrevUMI	GTCAGATGTGTATAAGAGACAGNNNNNNTTGACCAGGCAGCCCAGGG	
IgArevUMI	GTCAGATGTGTATAAGAGACAGNNNNNNGCCCTGGACCAGGCAKGCG	
IgMrevUMI	GTCAGATGTGTATAAGAGACAGNNNNNNTGCGAGGCAGCCAACGGCC	
Read1 primer	TCGTCGGCAGCGTCAGATGTGTATAAGAGACAG	V region amplification
Read2 primer	GTCTCGTGGGCTCGGAGATGTGTATAAGAGACAG	
N701	CAAGCAGAAGACGGCATACGAGATTCGCCTTAGTCTCGTGGGCTCGG	Illumina barcode sequences
S501	AATGATACGGCGACCACCGAGATCTACACTAGATCGCTCGTCGGCAGCGTC	
S502	AATGATACGGCGACCACCGAGATCTACACCTCTCTATTCGTCGGCAGCGTC	
S503	AATGATACGGCGACCACCGAGATCTACACTATCCTCTTCGTCGGCAGCGTC	
S504	AATGATACGGCGACCACCGAGATCTACACAGAGTAGATCGTCGGCAGCGTC	
S505	AATGATACGGCGACCACCGAGATCTACACGTAAGGAGTCGTCGGCAGCGTC	
S506	AATGATACGGCGACCACCGAGATCTACACACTGCATATCGTCGGCAGCGTC	
S507	AATGATACGGCGACCACCGAGATCTACACAAGGAGTATCGTCGGCAGCGTC	
S508	AATGATACGGCGACCACCGAGATCTACACCTAAGCCTTCGTCGGCAGCGTC	
