# Supplementary material for: Defining the structural basis for human alloantibody binding to human leukocyte antigen allele HLA-A*11:01
Source: Nat Commun. 2019 Feb 21;10:893. doi: 10.1038/s41467-019-08790-1 (PMC6385295; doi:10.1038/s41467-019-08790-1)
Supplement: Supplementary file 2 — Reporting Summary [file 41467_2019_8790_MOESM2_ESM.pdf]

nature research

Corresponding author(s): Paul A. MacAry

Reporting Summary

Nature Research wishes to improve the reproducibility of the work that we publish. This form provides structure for consistency and transparency in reporting. For further information on Nature Research policies, see Authors & Referees and the Editorial Policy Checklist.

Statistical parameters

When statistical analyses are reported, confirm that the following items are present in the relevant location (e.g. figure legend, table legend, main text, or Methods section).

n/a	Confirmed	
☐	☒	The exact sample size (*n*) for each experimental group/condition, given as a discrete number and unit of measurement
☐	☒	An indication of whether measurements were taken from distinct samples or whether the same sample was measured repeatedly
☐	☒	The statistical test(s) used AND whether they are one- or two-sided<br>*Only common tests should be described solely by name; describe more complex techniques in the Methods section.*
☒	☐	A description of all covariates tested
☐	☒	A description of any assumptions or corrections, such as tests of normality and adjustment for multiple comparisons
☐	☒	A full description of the statistics including central tendency (e.g. means) or other basic estimates (e.g. regression coefficient) AND variation (e.g. standard deviation) or associated estimates of uncertainty (e.g. confidence intervals)
☐	☒	For null hypothesis testing, the test statistic (e.g. *F*, *t*, *r*) with confidence intervals, effect sizes, degrees of freedom and *P* value noted<br>*Give P values as exact values whenever suitable.*
☒	☐	For Bayesian analysis, information on the choice of priors and Markov chain Monte Carlo settings
☒	☐	For hierarchical and complex designs, identification of the appropriate level for tests and full reporting of outcomes
☒	☐	Estimates of effect sizes (e.g. Cohen's *d*, Pearson's *r*), indicating how they were calculated
☐	☒	Clearly defined error bars<br>*State explicitly what error bars represent (e.g. SD, SE, CI)*

Our web collection on statistics for biologists may be useful.

Software and code

Policy information about availability of computer code

Data collection	No software was used for data collection.
Data analysis	MIXCR (v2.0.2) was used for analyzing the next-generation sequencing results of B cell; briefly, to define complementarity-determining regions (CDRs) and framework regions, and germline genes and cluster reads likely to originate from the same antibody clone. The structural data set was processed with XDS and XscALE (version Jan 26 2018). The structure of the complex protein was determined by molecular replacement using PHASER-MR within the PHENIX software package (version 1.13-2998-000), and using the PDB code: 1W72 as a search probe. A model for the 3D structure of the complex HLA-A*11:01 with 2E3-Fab was built iteratively at the computer graphics using COOT (0.8.9.1), and refined using BUSTER (version 2.10.3). Structure visualization and modeling was performed using USCF Chimera (version 1.12) and Pymol (Open-source pymol 1.8.6.0).

For manuscripts utilizing custom algorithms or software that are central to the research but not yet described in published literature, software must be made available to editors/reviewers upon request. We strongly encourage code deposition in a community repository (e.g. GitHub). See the Nature Research guidelines for submitting code & software for further information.

Data

Policy information about availability of data

All manuscripts must include a data availability statement. This statement should provide the following information, where applicable:

- Accession codes, unique identifiers, or web links for publicly available datasets
- A list of figures that have associated raw data
- A description of any restrictions on data availability

The datasets generated during X-ray crystallography are available in the worldwide Protein Data Bank (wwPDB) repository, under accession code 6ID4.
The data that support the findings of next-generation sequencing of patient B cells are deposited to figshare under DOI 10.6084/m9.figshare.7618520.
All other data generated or analysed during this study are included in this published article (and its supplementary information files).
Source data for Fig. 1a, Fig. 1b, Fig. 4b, Fig. 4c, Fig. 4d, Fig. 4e, Fig. 5a, Fig. 5b, Fig. 5c, Fig. 5d, Supplementary Fig. 1b, Supplementary Fig. 1c, and Supplementary Fig. 1d are provided with the paper.

Field-specific reporting

Please select the best fit for your research. If you are not sure, read the appropriate sections before making your selection.

☒ Life sciences ☐ Behavioural & social sciences ☐ Ecological, evolutionary & environmental sciences

For a reference copy of the document with all sections, see nature.com/authors/policies/ReportingSummary-flat.pdf

Life sciences study design

All studies must disclose on these points even when the disclosure is negative.

Sample size	No sample size-based calculation was performed. Experiments were repeated at least three times where applicable. The exact number of repeats are provided in the figure legends. Antibody binding was tested against 20 different Epstein-Barr virus-transformed B lymphoblastoid cell lines (EBV-BCLs) that express common Asian HLA Class I types.
Data exclusions	No data has been excluded.
Replication	Please note that the Clinical prognostic test on a patient sample was an official clinical test performed by the Health Sciences Authority (Singapore) laboratory that is internationally accredited to perform this test. This experiment was therefore not replicated.<br>The clinical prognostic test on monoclonal antibody 2E3 was also performed by the Health Sciences Authority (Singapore) laboratory. It was replicated and the reactive HLA Class I alleles identified were the same. Differences in the exact mean fluorescence intensity (MFI) values were expected because this is not a quantitative assay.<br>ELISA, kinetic study, flow cytometry, and antibody-dependent cell-mediated cytotoxicity (ADCC) assays were successfully repeated 3 times.<br>Complement-dependent cytotoxicity (CDC) assays were successfully repeated 4 times.<br>Sorting of human participant peripheral blood mononuclear cells (PBMCs) and subsequent next-generation sequencing was not repeated due to sample availability and the nature of the experiment.
Randomization	Not relevant (study does not involve allocation to experimental groups)
Blinding	Not relevant (study does not involve allocation to experimental groups)

Reporting for specific materials, systems and methods

Materials & experimental systems

n/a	Involved in the study	
☐	☒	Unique biological materials
☐	☒	Antibodies
☐	☒	Eukaryotic cell lines
☒	☐	Palaeontology
☒	☐	Animals and other organisms
☐	☒	Human research participants

Methods

n/a	Involved in the study	
☒	☐	ChIP-seq
☐	☒	Flow cytometry
☒	☐	MRI-based neuroimaging

Unique biological materials

Policy information about availability of materials

Obtaining unique materials	PBMCs were obtained from human research participants. EBV-BCLs were obtained from the Department of Microbiology and Immunology, National University of Singapore. Monoclonal antibody 2E3 in all antibody/antibody fragment forms are produced in-house by P.A.M. lab.

Antibodies

Antibodies used	W6/32 (purified from hybridoma culture supernatant, ATCC, #HB-95), 2E3-IgG1 (produced in-house), 2E3-IgG2 (produced in-house), 2E3-IgG3 (produced in-house), 2E3-IgG4 (produced in-house), 2E3-chimeric antibody with mouse IgG1 Fc (produced in-house)<br>goat anti-human IgG HRP conjugate (Thermo Fisher Scientific, #31413, lot #RE2206953), goat anti-mouse IgG Fc antibody HRP conjugate (Thermo Fisher Scientific, #31439, lot #SC2357664), goat-anti-mouse IgG antibody AF488 conjugate (Life Technologies, #A11029, lot #1745855), anti-6X his tag antibody (Abcam #ab18184), mouse-anti-human CD27 AF647 conjugate (AbD Serotec #MCA755A647), mouse-anti-human CD19 Pacific Blue conjugate (AbD Serotec #MCA1940PB), goat-anti-human IgM AF488 conjugate (Life Technologies, #H15101, lot # 1875778A), anti-M13 antibody HRP conjugate (GE Healthcare, #27-9421-01, lot #9505526)
Validation	In-house antibody binding to relevant HLA Class I was confirmed by results presented in the manuscript. Validity statements for commercial antibodies are provided on the manufacturers' websites.

Eukaryotic cell lines

Policy information about cell lines

Cell line source(s)	Twenty different EBV-BCLs were obtained from the Department of Microbiology and Immunology, NUS. The EBV-BCLs are named as follow: WGP060, CM376, CF890, CF1096, CM357, CF986, CF960, CF863, CM374, CM774, CM956, CM957, CF493, CF515, CF886, CF1094, WGP006, WGP014, WGP036, and WGP047.
Authentication	None of the cell lines used were authenticated.
Mycoplasma contamination	Cell lines were not tested for mycoplasma contamination.
Commonly misidentified lines<br>(See ICLAC register)	N.A.

Human research participants

Policy information about studies involving human research participants

Population characteristics	Use of PBMCs from one human research participant.<br>Biospecimen type: PBMCs<br>Disease status of patient: with a functioning kidney allograft after renal transplantation<br>Vital state of patient: alive<br>Clinical characteristics of patient: female, 66 years old, Chinese<br>Clinical diagnosis of patient: with a functioning allograft after deceased donor renal transplant at National University Hospital in Sep 2010<br>Sample collection mechanism: consented to 45ml of blood draw, of which 24ml is converted to PBMC.<br>Type of long-term preservation: Storage in liquid nitrogen.<br>Constitute of preservative: Freezing media (Filtered/Heat inactivated 90%FBS [Hyclone SH30070.03] + 10%DMSO [Sigma D2650-100ml]).<br>Storage temperature: -180°C<br>Storage duration: up to 15 years after collection. Specimen was collected for patient on 10 Nov 2016 and had been stored for 2 years (drawn 19 Nov 2018) at time of analysis<br>Shipping temperature: -78.5°C (dry ice)
Recruitment	The study was approved by the National Healthcare Group Domain Specific Review Board (NHG DSRB) which is accredited by the Association for the Accreditation of Human Research Protection Program (AAHRPP). The subject recruited in this study was recruited as part of a larger study to evaluate Anti HLA antibody specificity and antibody characteristics among prevalent renal transplant recipients on follow-up at the National University Centre for Organ Transplantation (NUCOT). Eligible subjects were approached for consent to participate in this study when they were seen in the NUCOT clinic for their regular clinic visit. The main components of the study protocol were: 1. Collection of 45 ml of blood; 2. Identification of Anti HLA antibody specificity from testing of serum samples from enrolled patients; 3. Collection of relevant clinical, laboratory and immunological information on enrolled patients with and without Anti HLA antibodies; 4. Analysis of Anti HLA specificity of antibodies by the Single Antigen Bead Luminex assay performed at an American Society for Histocompatibility and Immunogenetics (ASHI) certified laboratory in Singapore (HSA); 5. Identification of donor specificity of Anti HLA antibodies by examination of Donor and Recipient HLA typing.<br><br>Potential subjects were given the option of declining participation in this study with the assurance that their care would not be

affected. Blood collection will be performed at the next clinic visit so as to give additional time for the patients to withdraw from the study if they wished to do so. Subjects had the information given in a language they could understand with appropriate translation as required. All consents were witnessed by a third party and study procedures performed only after written and informed consent was obtained.

Inclusion criteria:
1. Adult kidney transplant recipients ie. between the ages 21 and 90 years old
2. Asian race/ethnicity
3. Have received a documented deceased or live donor kidney transplant and are currently on follow-up at the National University Centre for Organ Transplantation (NUCOT) in NUH, and who agree to provide an additional 45ml of blood for research purposes.

Exclusion criteria:
1. Intercurrent Illness
2. Pregnancy (clinically confirmed)
3. Unable to give informed consent
4. Kidney transplant from an Undocumented overseas donor

The following possible sources of bias were considered in this study
1. The study design only included prevalent transplant patients with a functioning allograft. Hence those with a particularly pathogenic anti HLA antibody that led to graft loss would have been excluded.
2. All potential subjects were given information in a language they could understand and were given the opportunity to decline participation. All potential subjects were also given time to consider the study proposal and had their blood taken for the purpose of the study at a subsequent visit and were allowed to withdraw consent at any time. Assurance of care by the clinicians at NUCOT was given regardless of provision of consent. All consents were obtained in the presence of a third party. With this study design, it is possible that patients who declined participation may have done so due to unique socio-cultural characteristics that prevented them from understanding the nature of the study and with complying to clinical treatments. While these could potentially lead to non-adherence and the development of Anti HLA antibodies, these inter-relationships were deemed to be minimal and expected to have minimal impact on this study.
3. Exclusion of kidney transplants from undocumented donors could have introduced some bias as NUCOT is a tertiary referral institution and a proportion of the prevalent patients would have been necessarily excluded. However this was necessary so as to include only those cases whose donor and recipient HLA typing was available so that the specificity of the Anti HLA antibody could be identified and so as to clearly identify Donor Specificity.

Hence, bias if present was deemed to be largely minimized by ensuring a rigorous consent process.

Flow Cytometry

Plots

Confirm that:

☒ The axis labels state the marker and fluorochrome used (e.g. CD4-FITC).

☒ The axis scales are clearly visible. Include numbers along axes only for bottom left plot of group (a 'group' is an analysis of identical markers).

☒ All plots are contour plots with outliers or pseudocolor plots.

☒ A numerical value for number of cells or percentage (with statistics) is provided.

Methodology

Sample preparation

Cell surface binding assay: in-house EBV-BCLs were washed, stained with relevant antibodies at 4°C for 1 h, protected from light. Cells were then washed in staining buffer (PBS supplemented with 1% fetal bovine serum)and acquired.
CDC assay: Target cells were incubated with antibody of interest for 1h, followed by a 3 h incubation with 10% baby rabbit complement serum (Cedarlane, #CL3441). Cells were washed in 1x PBS, stained with sytoxgreen and resazurin (ThermoFisher Scientific, #L34951) for 15 min, protected from light. Cells were kept in a cell incubator for all incubation steps.
ADCC assay: PBMCS from healthy donors were isolated and Natural Killer cells were enriched for via magnetic beads-based negative selection (STEMCELL #19055). NK cells were stimulated with 1500 units/mL IFN-alpha (ThermoFisher Scientific, #PHC4814) and cultured overnight at 37°C. Target cells were labelled with 1000x diluted CFSE (ThermoFisher Scientific, #C34564) and incubated with antibody of interest for 1 h. NK cells were then added (at a ratio of NK cells : target cells = 4:1) to the target cells for 4 h incubation. Cells were kept in a cell incubator for all incubation steps. Cells were then washed with staining buffer and labeled with 7AAD (ThermoFisher Scientific, #A1310) at a final concentration of 20 ug/mL. EBV-BCLs were washed and co-incubated with the relevant antibodies at 37°C. Cells were then stained with markers for viability for 15 minutes, washed and acquired.
FACS sorting: PBMCs from a kidney transplant recipient were resuspended in staining buffer and stained with mouse-anti-human CD27-AF647, mouse-anti-human CD19-PB, and goat-anti-human IgM-AF488 for 1 hour at 4°C, protected from light. Cells were washed, resuspended, and stained with propidium iodide (Sigma-Aldrich #P4864-10ML) at a final concentration of 10 ug/mL.

Instrument

Flow cytometry staining: Attune NxT acoustic focusing cytometer (Life technologies)
Cell sorting: BD FACSAria Fusion (BD Biosciences)

Software

FlowJo VX

Cell population abundance	A total cell population of at least 5000 events was acquired and recorded except when extremely high percentage of cell death was expected (e.g. upon treatment with TritonX or saponin).
Gating strategy	Cell surface binding assay: single cells were gated using FSC-A and FSC-H. Lymphocytes were gated using FSC-A and SSC-A. Cells positive for AF488-mouse IgG (FC) on histogram were defined as positive for antibody binding.<br>CDC assay: single cells were gated on using FSC-A and FSC-H. Cells that are Sytoxgreen-high and resorufin-mid were gated as the "dead" population. Cells that are Sytoxgreen-low and resorufin-high were gated as the "healthy" population.<br>ADCC assay: target cells were gated on the CFSE-positive population. Single cells were gated on using FSC-A and FSC-H. Cells that are 7AAD-high and FSC-A-low were defined as the "dead" population.<br>FACS sorting: lymphocytes were gated on using FSC-A and SSC-A. Single cells were gated on using FSC-A and FSC-H. Cells that are PI-negative were gated as live cells. Single cells were confirmed using SSC-A and SSC-H. PB-CD19-positive population was defined as B cells. CD19-positive cells were sorted into four tubes based on the four quadrants using FITC-IgM and AF647-CD27.

☒ Tick this box to confirm that a figure exemplifying the gating strategy is provided in the Supplementary Information.
